# High Serum Anti-Müllerian Hormone Concentrations Are Associated With Poor Pregnancy Outcome in Fresh IVF/ICSI Cycle but Not Cumulative Live Birth Rate in PCOS Patients

**DOI:** 10.3389/fendo.2021.673284

**Published:** 2021-05-26

**Authors:** Yaxin Guo, Shuai Liu, Shiqiao Hu, Fei Li, Lei Jin

**Affiliations:** Reproductive Medicine Center, Tongji Hospital, Tongji Medical College, Huazhong University of Science and Technology, Wuhan, China

**Keywords:** anti-Müllerian hormone, assisted reproduction, polycystic ovary syndrome, live birth rate, cumulative live birth rate, clinical pregnancy rate, normal fertilization rate

## Abstract

**Objective:**

To investigate the association between baseline serum Anti-Müllerian hormone (AMH) levels and IVF/ICSI outcomes in women with polycystic ovary syndrome (PCOS).

**Design:**

Retrospective study.

**Setting:**

Reproductive medicine center in a hospital.

**Population:**

2436 PCOS patients (Rotterdam criteria) who underwent their first fresh IVF/ICSI cycles were divided into three groups on the basis of the <25th (Group 1, n=611), 25 to 75th (Group 2, n=1216), or >75th (Group 3, n=609) percentile of baseline serum AMH level.

**Intervention(s):**

Baseline serum AMH levels measured on the 2-3 days of spontaneous menstrual cycle before IVF/ICSI treatment.

**Main Outcome Measure(s):**

Live birth rate (LBR), cumulative live birth rate (CLBR), clinical pregnancy rate (CPR), and normal fertilization rate (FR).

**Result(s):**

The LBR, CPR, and FR were significantly increased in Group 1 than Group 2 and Group 3, however, CLBR was similar between the three groups. The LBR were 46.6%, 40.5%, and 39.4% in Group 1, Group 2, and Group 3 respectively. The CPR were 53.0%, 47.0%, and 45.5%, respectively. The FR was highest in Group 1 (61.7%, P<0.05), but there was no uniform reverse trend with the AMH level. CLBR were 68.7%, 70.4%, and 71.3%, respectively. Although women in Group 1 were older (p < 0.05) and had higher body mass index (BMI) (p < 0.05), binomial logistic regression analysis used age, BMI, FSH, and AMH as independent variables indicated that only AMH was significantly associated with LBR and CPR. Nevertheless, binomial logistic regression analysis used age, BMI, FSH, AMH, and the number of retrieved oocytes as independent variables indicated that only the number of retrieved oocytes was significantly correlated with CLBR. After stratifying by age, the negative relationship between baseline AMH level and LBR and CPR remained only in the patients <30 years old.

**Conclusion(s):**

Higher baseline AMH level in PCOS women resulted in lower LBR, CPR, and FR but did not influence CLBR.

## Introduction

Anti-Müllerian hormone (AMH) is a dimeric glycoprotein belonging to the transforming growth factor β family. AMH is secreted by the granulosa cells (GCs) of small antral follicles (1, 2). AMH is not only one of the most effective predictors of ovarian reserve, it is also widely considered to reflect the ovarian response in assisted reproductive technology (ART) (3–5). Timely reduction of AMH at the time of follicle selection contributes to normal follicle development and ovulation (6).

Polycystic ovary syndrome (PCOS) is the most common endocrine disorder, affecting 10 to 20% of reproductive-aged women worldwide (7, 8). It is the main cause of infertility resulting from hyperandrogenism and oligo-anovulation (7). However, the complex pathogenesis of PCOS is still poorly understood. There is evidence that increased serum levels of AMH may play a part in disordered folliculogenesis in PCOS (9), which is due to the joint effect of an increase in the number of follicles and excessive AMH production by each follicle (10, 11). In addition, studies have found a possible relationship between AMH levels and the severity of PCOS (12–14), which may confuse the predictive value of AMH on ovarian reserve and pregnancy outcomes in PCOS women undergoing ART. The few studies that have evaluated the relationship have reported inconsistent results, with some showing a positive relationship between the AMH concentrations and reproductive outcomes in PCOS patients (15, 16), and others suggesting the opposite results (17, 18). Other studies have tried to assess the association based on different PCOS phenotypes (19–21), but there was no consistent conclusion. Furthermore, the sample size of these studies (15–21) was limited. With the extensive use of highly efficient embryo cryopreservation technology, cumulative live birth rate (CLBR) encompassing live birth outcomes in the fresh and subsequent frozen embryo transfer (FET) following a single ovarian stimulation cycle is progressively regarded as a significant indicator for assessing the success of ART but so far, no study has directly explored the relationship of CLBR and baseline AMH level in PCOS patients. Therefore, the objective of the study was to evaluate the relationship between baseline serum AMH levels and reproductive outcomes in a larger PCOS population undergoing *in vitro* fertilization (IVF) or intracytoplasmic sperm injection (ICSI), especially to discuss the direct relationship of CLBR and baseline AMH levels.

## Materials and Methods

### Patients

We retrospectively reviewed 2436 PCOS patients undergoing first fresh IVF/ICSI cycles with autologous oocytes at the Reproductive Medicine Center of Tongji Hospital between January 1, 2016, and December 31, 2019. According to the Rotterdam criteria established in 2004, patients with PCOS are diagnosed by the presence of at least two of the three following symptoms: clinical and/or biochemical signs of hyperandrogenism, anovulation (oligo- or amenorrhea), and/or polycystic ovaries (22). No participants were diagnosed with congenital adrenal hyperplasia, Cushing’s syndrome, or androgen-secreting tumors. Patients enrolled in an oocyte donation program and undergoing preimplantation genetic diagnosis (PGD) or preimplantation genetic screening (PGS) were also excluded. We extracted data from electronic medical records.

Women were divided into three groups on the basis of the <25th (Group 1, n=611), 25 to 75th (Group 2, n=1216), or >75th (Group 3, n=609) percentile of baseline serum AMH level.

### Ovarian Stimulation and IVF Procedures

Patients were subjected to an individualized controlled ovarian stimulation protocol according to ovarian reserve testing and other characteristics. All enrolled patients received GnRH agonist or antagonist treatment. Details on the ovarian stimulation protocol have been previously described (23) and oocytes were retrieved transvaginally 34–36 hours after hCG injection (24, 25). Oocyte maturation rate was defined as the number of MII oocytes divided by the number of retrieved oocytes.

Fertilization was performed either by conventional insemination or ICSI. Normal fertilization was defined as zygotes with two pronuclei (2PN). The normal fertilization rate was defined as the number of 2PN divided by the number of retrieved oocytes in IVF or 2PN divided by the number of MII in ICSI. All of the embryos were checked on the morning of day 3 after oocyte retrieval. Fewer than two embryos with the highest score were selected for transfer on day 3, and excess embryos were cryopreserved or continuously cultured to the blastocyst stage. According to local criteria, elective freezing of all embryos was considered when the patient was at risk of ovarian hyperstimulation syndrome (OHSS), had an unsuitable endometrial environment or premature progesterone elevation, or other personal circumstances in which fresh-embryo transfer was not preferred. Surplus embryos were cryopreserved on the day of the embryo transfer by vitrification using the Cryotop system (26). Details of the embryo cryopreservation and frozen-thawed embryo transfer protocols have been previously described (23).

### AMH Assay

Baseline serum AMH concentration was determined by an enzyme-linked immunosorbent assay (ELISA, AMH ELISA kit; Kangrun Biotech, China) on the 2-3 days of spontaneous menstrual cycle before IVF/ICSI treatment.

### Main Outcome Measures

The primary outcomes include live birth rate (LBR) and cumulative live birth rate (CLBR) per aspiration, and the secondary outcomes include the clinical pregnancy rate (CPR) and normal fertilization rate (FR). Live birth was defined as the delivery of one or more live infants. LBR was defined as the number of live births divided by the number of women in a group. Cumulative live birth was defined as live birth that occurs during the fresh cycle and the subsequent FET cycle after the same ovarian stimulation cycle until one live birth occurred or all embryos were used or December 31, 2019. Considering that the frozen embryos of patients who underwent the fresh cycle in 2019 have not yet been completely transplanted, these patients were excluded from the analysis of CLBR. Clinical pregnancy was defined as ultrasonographic visualization of one or more gestational sacs. The available embryo rate was the number of day three available embryos divided by the number of retrieved oocytes.

### Statistical Analysis

The primary analysis of the study consisted of a comparison of the main baseline characteristics, IVF/ICSI cycle characteristics, and pregnancy outcomes between the three groups. Categorical data are expressed as a corresponding percentage and the number of cases while continuous data are presented by mean ± standard deviation (SD).

The categorical variables were compared by the chi-squared test while the continuous variables were compared by the one-way analysis of variance test (Bonferroni correction in post-hoc test) when the data had a normal distribution and homogeneity of variance. The Kruskal-Wallis test was performed in the case of non-normal or Heterogeneity of variance.

To exclude the influence of confounding factors such as age and body mass index (BMI), the binomial logistic regression analysis was conducted in a forward manner. In addition to this, receiver operating characteristic (ROC) curves were generated to investigate the predictive value of Day-3 serum AMH level for fresh LBR, CPR.

Data analysis was performed by use of SPSS for Windows, version 23 (SPSS Inc., Chicago, IL). All significance tests were 2-tailed and P < 0.05 was noted to be statistically significant.

## Results

The main baseline characteristics in the three groups are shown in **Table 1**. Enrolled women were PCOS patients between 20 and 44 years old. The mean ( ± SD) value of baseline serum AMH concentration for all 2436 patients was 10.57 ± 4.66 ng/ml, ranging from 0.94 to 25.00 ng/ml.

**Table 1:** Baseline characteristics.

Variable	Day-3 Serum AMH (ng/ml)			P value		
	Group 1, AMH ≤ 6.77 N=611	Group 2, 6.77-14.30 n=1216	Group 3, AMH≥14.30 N=609	p-value Group 1 *vs.* Group 3	p-value Group 1 *vs.* Group 2	p-value Group 2 *vs.* Group 3
Maternal age, y	29.50 ± 3.72	28.88 ± 3.35	28.59 ± 3.14	<0.001	0.001	NS
Body mass index, kg/m^2^	23.31 ± 3.50	22.66 ± 3.22	22.38 ± 3.12	<0.001	0.001	NS
Baseline FSH, mIU/mL	6.80 ± 1.74	6.60 ± 1.56	6.53 ± 1.42	0.017	0.033	NS
Antral follicle count (AFC)	21.08 ± 4.31	23.24 ± 4.24	26.09 ± 6.94	<0.001	<0.001	<0.001
Duration of infertility, years	3.49 ± 2.31	3.46 ± 2.17	3.61 ± 2.24	NS	NS	NS
Infertility diagnosis				NS	NS	NS
Primary infertility, %	74.1 (453/611)	73.7 (896/1216)	75.0 (457/609)			
Secondary infertility, %	25.9 (158/611)	26.3 (320/1216)	25.0 (152/609)			
Infertility etiology, %				NS	NS	NS
Male factor	22.6 (138/611)	26.0 (316/1216)	22.0 (134/609)			
Tubal factor	36.8 (225/611)	40.3 (490/1216)	38.8 (236/609)			
Endometriosis	3.4 (21/611)	2.2 (27/1216)	2.5 (15/609)			
Ovulatory	0.7 (4/611)	0.1 (1/1216)	0.3 (2/609)			
Uterine malformation	4.6 (28/611)	4.6 (56/1216)	4.9 (30/609)			
Unexplained/Other	31.9 (195/611)	26.8 (326/1216)	31.5 (192/609)			

NS, not statistically significant.

In the current study, AMH levels decreased with increasing age, BMI, and serum FSH. As shown, antral follicle count (AFC) increased significantly from Group 1 to Group 2 to Group 3. The duration of infertility, Infertility diagnosis (primary or secondary infertility) and infertility etiology of women were comparable in the three groups (**Table 1**).

The cycle characteristics and ART outcomes are listed in **Table 2**. There were 1898 patients with a fresh ET. In total, 1790 patients were enrolled between 2016 and 2018, and all were considered in the analysis of CLBR, of which 1371 patients had fresh ET performed (367 couples chose fresh and then FET), 329 patients underwent freeze all and only FET and 90 patients did not undergo ET until December 31, 2019, due to no available embryos or for personal reasons.

**Table 2:** Cycle characteristics and ART outcomes.

Variable	Day-3 Serum AMH (ng/ml)			P value		
	Group 1, ≤6.77 N=611	Group 2,6.77-14.30 n=1216	Group 3, ≥14.30 N=609	p-value Low *vs.* High	p-value Low *vs.* Average	p-value Average *vs.* High
Intracytoplasmic sperm injection, %	29.5 (180/611)	31.2 (379/1216)	28.7 (175/609)	NS	NS	NS
Ovarian stimulation protocols				0.044	NS	NS
GnRH Antagonist, %	11.8 (72/611)	13.2 (160/1216)	16.6 (101/609)			
GnRH agonist, %	88.2 (539/611)	86.8 (1056/1216)	83.4 (508/609)			
Gonadotropin dose, IU	2116 ± 782	1863 ± 783	1740 ± 785	<0.001	<0.001	<0.001
E2 on hCG, pg/mL	2810 ± 1670	3561 ± 2105	4349 ± 2841	<0.001	<0.001	<0.001
P on hCG day, ng/mL	0.84 ± 0.48	0.86 ± 0.57	0.90 ± 0.65	NS	NS	NS
No. of oocytes retrieved	13.97 ± 5.81	16.00 ± 6.52	16.84 ± 8.04	<0.001	<0.001	NS
No. of MII oocytes	12.21 ± 5.32	14.01 ± 5.95	14.83 ± 7.58	<0.001	<0.001	NS
Oocyte maturation rate	0.8786 ± 0.1333	0.8795 ± 0.1325	0.8801 ± 0.1325	NS	NS	NS
Endometrial thickness, mm	11.96 ± 2.64	11.95 ± 2.63	11.78 ± 2.40	NS	NS	NS
No. of >14 mm follicles	12.91 ± 4.58	14.84 ± 4.90	16.68 ± 6.12	<0.001	<0.001	<0.001
Available embryo rate	0.6552 ± 0.1930	0.6466 ± 0.1993	0.6418 ± 0.2197	NS	NS	NS
No. of embryos transferred in fresh ET	1.20 ± 0.67	1.08 ± 0.70	0.90 ± 0.73	<0.001	<0.001	0.001
Live birth rate, %	46.6 (285/611)	40.5 (492/1216)	39.4 (240/609)	0.031	0.034	NS
Cumulative live birth rate, %	68.7 (272/396)	70.4 (643/914)	71.3 (342/480)	NS	NS	NS
Clinical pregnancy rate, %	53.0 (324/611)	47.0 (571/1216)	45.5 (277/609)	0.025	0.043	NS
Normal fertilization rate, %	61.7 (5266/8534)	59.9 (11664/19462)	59.9 (6144/10254)	0.038	0.016	NS

NS, not statistically significant.

The total consumption of gonadotrophins progressively decreased from Group 1 to Group 2 to Group 3. Conversely, estradiol levels on the day of HCG administration, the number of retrieved oocytes, metaphase II oocytes, and > 14 mm follicles were significantly increased with increasing serum AMH, while progesterone levels on the day of HCG administration showed no significant difference (**Table 2**). On average, approximately one fresh embryo was transferred for each group. The mode of fertilization (IVF or ICSI), oocyte maturation rate, available embryo rate, and endometrial thickness were similar in the three groups. Although the ratio of GnRH Antagonist treatment showed a gradual increase from Group 1 to Group 2 to Group 3 in our study, a high-quality meta-analysis (27) showed that GnRH antagonists do not seem to compromise ongoing pregnancy rates.

In our cohort, there was a significant increase in fresh LBR in PCOS women in Group 1 (46.6%) compared to Group 2 (40.5%) and Group 3 (39.4%). Similarly, Fresh CPR was significantly higher in Group 1 (53.0%) compared to Group 2 (47.0%) and Group 3 (45.5%). The FR was highest in Group 1 (61.7%, P<0.05), but there was no uniform reverse trend with the AMH level. However, there was no statistical difference in CLBR among the three groups, and even CLBR tended to increase with the increase of baseline AMH level.

**Figure 1:**
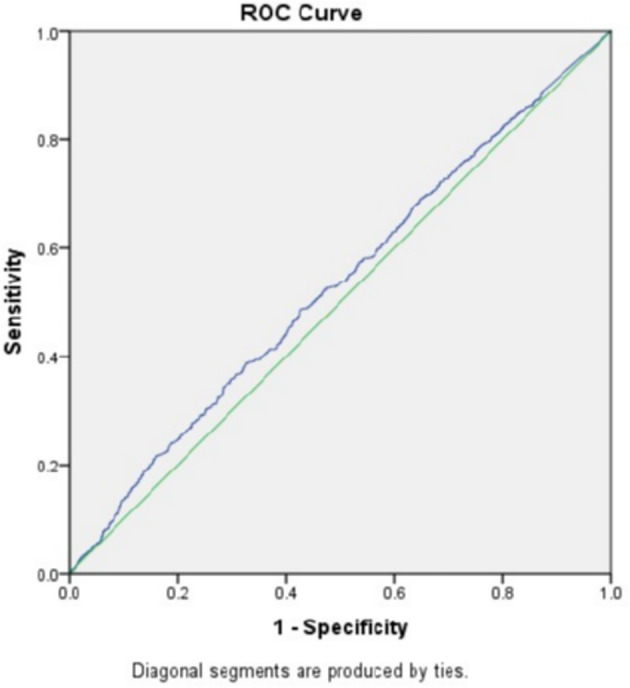
Receiver-operating characteristic (ROC) curve analysis for AMH as a predictor of LBR.

**Figure 2:**
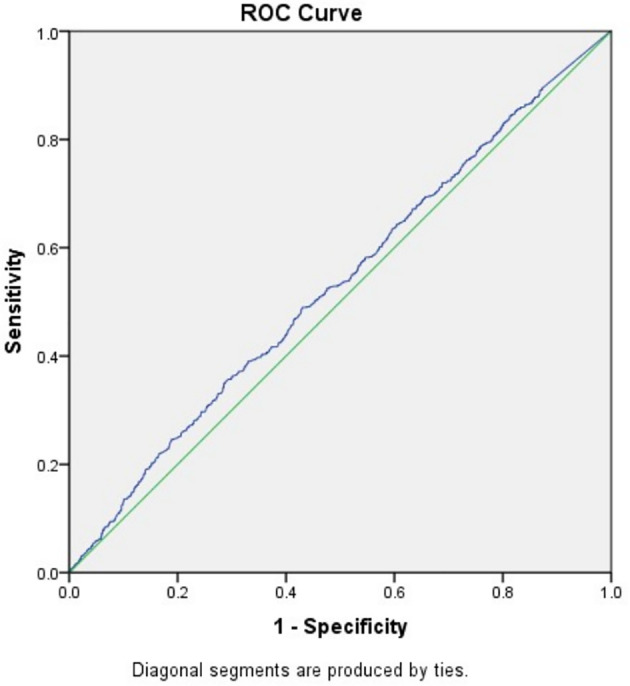
Receiver-operating characteristic (ROC) curve analysis for AMH as a predictor of CPR.

To exclude the influence of confounding factors, binomial logistic regression analysis used age, BMI, FSH, and AMH as independent variables were implemented and the result suggested that only AMH had a significant association with fresh LBR and CPR (data not shown). After that, the study went further to explore the predictive power of baseline AMH for fresh LBR by Receiver-operating characteristic (ROC) curve, which showed an area under the curve (AUC) of 0.531 (0.508–0.555, 95% confidence interval, P=0.008) (**Figure 1**), indicating overall poor predictive value. Another ROC curve was drawn for predicting CPR, the AUC was 0.531 (0.508–0.554, 95% confidence interval) (**Figure 2**), which also showed overall poor predictability. Moreover, Binomial logistic regression analysis used age, BMI, FSH, AMH, and the number of retrieved oocytes as independent variables and indicated that only the number of retrieved oocytes had a significant association with CLBR (data not shown).

Subgroup analysis was performed by stratifying women into <30 years, 30-35 years, and ≥35 years (**Supplemental Table 1**). In the patients <30 years old, there was a significant increase in fresh LBR in Group 1 (49.1%) compared to Group 2 (41.4%) and Group 3 (38.1%). Similarly, CPR was significantly higher in Group 1 (55.0%) compared to Group 2 (47.3%) and Group 3 (44.6%). But there was no significant difference in LBR and CPR between groups in patients 30-35 years and ≥35 years. Consistent with the entire population, CLBR was comparable in three groups in all age subgroups.

## Discussion

This is the first study to reveal a negative correlation between AMH and fresh CPR and FR in PCOS patients. This is the first time a study has directly explored the relationship between AMH levels and CLBR.

In our cohort, age decreased from women with low baseline AMH levels to women with average and high baseline AMH levels, which is in accordance with previous studies (28, 29) In the same way, BMI was significantly higher in women in Group 1, which is in agreement with previous studies indicating that there is a negative association between AMH and BMI in PCOS women (30–32). Decreased AMH production by the follicle unit instead of hemodilution may contribute to reduced AMH with increasing BMI (31). There was an inverse relationship between serum FSH and AMH levels in PCOS patients, which has been reported in previous studies (11, 33, 34) and remains unexplained to date (35, 36). Our opinion is consistent with a previous study (36) in which it is not believed that the negative impact of FSH on the production of AMH was responsible for the phenomenon. In contrast, there is evidence showing the positive impact of FSH on the production of AMH in the case of congenital gonadotrophic insufficiency (37). Virginie Desforges-Bullet et al. (11) thought that impaired access of FSH to follicles possibly explains why the GCs in PCOS continues to produce elevated levels of AMH, which possibly explain the inverse relationship.

In agreement with previous studies (4, 38–41), the number of total oocytes, metaphase II oocytes, and >14 mm follicles were statistically different in groups in our study, verifying the positive predictive value of baseline AMH levels on the ovarian response to stimulation regimen. The total consumption of gonadotrophins progressively decreased from low to high baseline in the AMH group, which is consistent with previous data indicating that baseline AMH levels positively predict ovarian responsiveness to gonadotrophin in PCOS (15, 17, 18, 42). The estradiol (E2) level on the day of hCG administration was significantly positively correlated with serum AMH levels in this cohort. This differs from the results of previous studies (15, 17, 18). Although a study has suggested a positive relationship (18), another study has suggested an inverse relationship (15), and a recent literature review found that there was no correlation between AMH concentration and E2 level on the hCG day (17). E2 is synthesized by GCs through the action of aromatase, and many studies have shown that AMH can inhibit the expression of FSH- and LH-induced aromatase in GCs resulting in a significant reduction in E2 production (33, 35, 43), which seem to contradict our results. However, there are also studies showing that E2 and AMH are positively correlated (18, 42, 44, 45). It was speculated that lower AMH level in mature follicle compared to AMH in preantral and small antral follicles was responsible for this, which contributes to the relief of inhibition on aromatase expression and subsequent estradiol synthesis (18). Furthermore, studies have found that AMH is expressed exclusively by GCs with mitotic activity (43, 44). Therefore, an alternate explanation for our findings is that E2 may reflect the proliferation of GCs during FSH-stimulated follicle growth, which could neutralize AMH inhibition on FSH- induced aromatase activity (42).

In our study, PCOS women undergoing assisted reproduction have high AMH levels, and fresh LBR, CPR, and FR in the low baseline AMH women were significantly elevated than those with average or high baseline serum AMH levels. However, CLBR was not significantly different between the three groups and even tended to increase with the increase in AMH level. Given the fact that obesity and age have negative effects on reproductive outcomes (46), we performed a multivariable logistic regression analysis in a forward manner. Fresh LBR and CPR remained significantly correlated with AMH level after adjusting for age and BMI, while ROC curve analysis showed the poor predictive value of baseline AMH level. As for the multivariable logistic regression analysis for influencing factors of CLBR, only the number of retrieved oocytes was shown to be significantly correlated with it. After stratifying by age, the negative relationship between baseline AMH level and fresh LBR and CPR remained only in the patients <30 years old.

There have been four studies published that explore the relationship between baseline serum AMH level and pregnancy outcome following IVF/ICSI treatment in PCOS women (15–18). Our results contrast to these earliest studies (15), in which they enrolled 60 PCOS patients and reported fresh CPR and FR were significantly increased with AMH levels on day 3 of the stimulations cycle. On the contrary, Xi et al. (18) found the lowest CPR in PCOS women with high AMH levels, although this difference was not significant. Interestingly, the study by Tal et al. in 2014 (16) showed that increased AMH levels were correlated with greater ovarian stimulation and higher fresh CPR, but a recent study by Tal et al. (17) suggested that higher serum AMH levels have poorer LBR in a fresh cycle, which agrees with our study. No statistically significant correlation between CPR, FR, and AMH level was reported, possibly due to the limitation of sample size (17). This discrepancy among the four studies is unclear and may be attributed to the differences in the studied populations and sample size. As is well-known, PCOS is a heterogeneous syndrome and the concentration of AMH appears to be relevant to the severity of it. AMH has been observed to be higher in hyperandrogenic than in normal-androgenic PCOS women (32, 47, 48). Furthermore, in another study, anovulatory PCOS women have elevated serum AMH levels than ovulatory and hyperandrogenic PCOS women (19). Similarly, AMH levels in amenorrheic and oligomenorrheic PCOS patients were found to be higher than those in eumenorrheic PCOS patients (49), which possibly indicate a role for AMH in the pathogenesis of anovulation in PCOS women. Consequently, elevated AMH levels in PCOS women are correlated with disease severity (4, 35, 50) and may confuse its predictive effect on ovarian reserve, thus confounding the relationship between baseline AMH level and pregnancy outcomes in patients with PCOS (50). Notably, there are variable AMH concentration categorizations across studies and the range of AMH in our study is the widest, and overall AMH levels are higher.

As we all know, age is a significant factor influencing a woman’s ability to conceive (51–53). Given the wide range of maternal age in our study, patients were stratified according to age at oocyte retrieval. The negative relationship between baseline AMH level and fresh LBR and CPR remained only in the patients <30 years old. In the previous studies that consistently suggest this inverse relationship (17, 18), one included only patients under 35 years old, and the other enrolled patients 18 to 40 years old. Another study reported a positive relationship (15), but the age range of patients was not mentioned. Therefore, although the comparable CPR and LBR in patients older than 30 years old in our study may be due to the limitation of sample size in subgroups, the different age ranges between the studies were likely to be another factor that confuses the relationship between AMH and pregnancy outcomes in PCOS patients.

As for the explanation of the correlation between higher AMH and poor reproductive outcome in fresh cycle in PCOS women, firstly, AMH excess in GCs from PCO would be involved in the follicle excess of PCO (54–56) and the follicular arrest in anovulatory patients (33, 36), thus high AMH levels may result in the disturbed folliculogenesis of PCOS. In addition, as mentioned above, AMH levels are related to the severity of PCOS, and a positive link between AMH concentration and severity of hyperandrogenemia has been found (16, 32, 34, 57), which has a detrimental effect on oocyte quality and therefore contributes to lower fertilization rates (58). Moreover, a hyperandrogenic PCOS environment could compromise the endometrial homeostasis and receptivity (59), which could also partially explain the decline in fresh CPR. Women with high serum AMH levels are at increased risk of ovarian hyperstimulation and OHSS and therefore a higher risk of cycle cancellation. Furthermore, the high E2 level associated with ovarian hyperstimulation has been demonstrated to have harmful consequences on endometrial receptivity, which therefore affects embryo implantation (60, 61) and contributes to poor pregnancy outcomes in the fresh cycle.

However, notably, CLBR has a tendency to increase with the increase of AMH concentration. There is evidence suggesting that hyperandrogenic PCOS phenotypes confer significantly lower CLBR after ovarian stimulation and assisted reproductive technology compared with their normoandrogenic counterparts (20) and, as mentioned above, AMH levels showed a positive relationship with the degree of hyperandrogenemia (16, 32, 34, 57). In contrast, there are also data showing that CLBR was significantly higher in PCOS women with hyperandrogenemia when compared to PCOS women with normal androgen levels and non-PCOS patients with a PCO-like ovarian morphology, but the study was conducted under the background of in-vitro oocyte maturation (IVM) (62). Nevertheless, evidence has shown that CLBR had a positive relationship with the amount of retrieved oocytes in PCOS patients (63). In our study, the number of retrieved oocytes increased with the increase of baseline AMH level, and can thus partly explain the tendency of increased CLBR with the increase of AMH level. In addition, ovarian hyperstimulation and the associated high estrogen-induced endometrial receptivity damage no longer affects the FET cycle outcome. Although there are data showing that high AMH concentration may cause disturbed folliculogenesis in PCOS (33, 36, 54–56), our data suggested that higher AMH may have little effect on oocyte quality compared to medium high AMH level or the increase in oocyte production may counteract the decrease in oocyte quality. Further clinical studies and molecular biology experiments were needed to explore the possible impact of AMH on PCO folliculogenesis and oocyte quality. In addition, further studies including blastulation rates, blastocyst transfer and PGT-A could provide further information about oocyte quality and PCOS.

This study was large and most results have significant statistical differences, and the study also has other limitations. The major limitations of the study are attributed to its retrospective nature. However, the primary and secondary outcomes of the current study are objective indicators that are not affected by the subjective factors of patients. Moreover, residual confounding, such as different ovarian stimulation protocols and other unknown confounding factors potentially affect the strength of the association between baseline AMH levels and final pregnancy outcomes.

In conclusion, in the present cohort trial on 2436 PCOS women undergoing IVF/ICSI, we found that higher baseline AMH level resulted in lower fresh LBR, CPR, and FR and higher but not statistically significant CLBR compared to low and average AMH levels. The inconsistency between the fresh outcome and the cumulative live birth rate may be due to the increased risk of ovarian hyperstimulation caused by high AMH levels and the changes in endometrial receptivity and homeostasis associated with high AMH levels. Similar to the non-PCOS population, our study suggests that baseline serum AMH levels positively predict the ovarian response in PCOS patients and also that higher AMH may have little effect on oocyte quality compared to medium high AMH levels or an increase in oocyte production related to higher AMH, which may counteract the decrease in oocyte quality in PCOS patients. Larger, multicenter prospective studies and molecular studies are required to further clarify the relationship.

## Data Availability Statement

The raw data supporting the conclusions of this article will be made available by the authors, without undue reservation.

## Ethics Statement

The studies involving human participants were reviewed and approved by Medical Ethics Committee of Tongji Hospital, Tongji Medical College, Huazhong University of Science and Technology. The patients/participants provided their written informed consent to participate in this study.

## Author Contributions

LJ and FL conceived of and designed the study. YG and SL collected and analyzed the data and wrote the paper. SH gave some important suggestions for data analysis. All authors contributed to the article and approved the submitted version.

## Funding

This work was supported by the National Key R & D Program of China (2018YFC1002103) and the National Natural Science Foundation of China (No.81701521 to FL).

## Conflict of Interest

The authors declare that the research was conducted in the absence of any commercial or financial relationships that could be construed as a potential conflict of interest.

## Supplementary Material

Supplementary Table 1 Clinical data of patients in different age categories.
