# Supplementary material for: High Serum Anti-Müllerian Hormone Concentrations Are Associated With Poor Pregnancy Outcome in Fresh IVF/ICSI Cycle but Not Cumulative Live Birth Rate in PCOS Patients
Source: Front Endocrinol (Lausanne). 2021 May 26;12:673284. doi: 10.3389/fendo.2021.673284 (PMC8187895; doi:10.3389/fendo.2021.673284)
Supplement: Supplementary Table 1 — Clinical data of patients in different age categories. [file Table_1.docx]

# Supplementary Table 1 | Clinical data of patients in different age categories

| age | Group | N | AFC (n) | Oocyte yield (n) | Clinical pregnancy rate (%) | Live birth rate, % | Cumulative live birth rate, % |
| --- | --- | --- | --- | --- | --- | --- | --- |
| ≤30.0  (n=1720) | 1  2  3 | 387  876  457 | 21.42±3.98  23.36±4.35*  26.27±7.23* | 14.22±6.10  16.10±6.55*  16.95±8.11* | 55.0(213/387)  47.3(414/876)*  44.6(204/457)* | 49.1(190/387)  41.4(363/876)*  38.1(204/457)* | 71.5(186/260)  71.0(475/669)  70.6(255/361) |
| 30.0-35.0 (n=563) | 1  2  3 | 172  264  127 | 20.67±4.80  22.88±3.98*  25.69±6.18* | 13.75±5.26  15.93±6.56*  16.09±7.60 | 51.7(89/172)  45.8(121/264)  52.0(66/127) | 43.6(75/172)  38.3(101/264)  47.2(60/127) | 65.4(70/107)  70.7(133/188)  74.7(74/99) |
| ≥35.0  (n=153) | 1  2  3 | 52  76  25 | 19.85±4.69  22.99±3.81*  25.00±4.88* | 12.81±5.23  15.18±6.10  18.56±8.94* | 42.3(23/52)  47.4(36/76)  28.0(7/25) | 38.5(20/52)  36.8(28/76)  24.0(6/25) | 55.2(16/29)  61.4(35/57)  65.0(13/20) |

* Significant differences after Bonferroni correction between Group 1 and Group 2 or Group 3 (P < 0.05).
